# Outcomes of Witnessed Versus Unwitnessed Patients With Stroke After Endovascular Therapy in the Extended Time Window

**DOI:** 10.1161/STROKEAHA.125.052355

**Published:** 2025-11-12

**Authors:** Liisa Tomppo, Nicolas Martinez-Majander, Muhammad M. Qureshi, Thanh N. Nguyen, Raul G. Nogueira, Simon Nagel, Jelle Demeestere, Volker Puetz, Hilde Henon, Marta Olive-Gadea, João Pedro Marto, Anne Dusart, Peter A. Ringleb, Osama O. Zaidat, Diogo C. Haussen, Mahmoud H. Mohammaden, Mohamad Abdalkader, Jean Raymond, Santiago Ortega-Gutierrez, Sunil A. Sheth, Hiroshi Yamagami, João Nuno Ramos, Francois Caparros, Daniel P.O. Kaiser, Marc Ribo, Sergio Salazar-Marioni, Kanta Tanaka, Pekka Virtanen, Ajit S. Puri, James E. Siegler, Syed F. Zaidi, Mouhammad Jumaa, Eugene Lin, Jordi Mayol, Rita Ventura, Simon Winzer, Piers Klein, Flavio Bellante, Jorge Cespedes, Anke Wouters, Hesham E. Masoud, Liqi Shu, Alicia C. Castonguay, Christian Herweh, Monica Cheng, Wei Hu, Daniel Roy, Shadi Yaghi, Robin Lemmens, Charlotte Cordonnier, Markus A. Möhlenbruch, Daniel Strbian

**Affiliations:** Department of Neurology (L.T., N.M.-M., D.S.), Helsinki University Hospital, University of Helsinki, Finland.; Department of Radiology (P.V.), Helsinki University Hospital, University of Helsinki, Finland.; Department of Radiology, Radiation Oncology, Boston Medical Center, Boston University Chobanian and Avedisian School of Medicine, MA (M.M.Q.).; Department of Neurology, Radiology, Boston Medical Center, Boston University Chobanian and Avedisian School of Medicine, MA (T.N.N., P.K.).; Department of Neurology and Neurosurgery, University of Pittsburgh Medical Center, PA (R.G.N.).; Department of Neurology, Klinikum Ludwigshafen, Germany (S.N.).; Department of Neurology (S.N., P.A.R.), Heidelberg University Hospital, Germany.; Department of Neuroradiology (C.H., M.A.M.), Heidelberg University Hospital, Germany.; Department of Neurology, UZ Leuven, Belgium (J.D., A.W., R.L.).; Laboratory for Neurobiology, KU Leuven, Belgium (J.D., A.W., R.L.).; Department of Neurology, Faculty of Medicine and University Hospital Carl Gustav Carus, Technische Universität Dresden, Germany (V.P., S.W.).; Dresden Neurovascular Center, Faculty of Medicine and University Hospital Carl Gustav Carus, Technische Universität Dresden, Germany (V.P., D.P.O.K.).; Department of Neurology, Centre Hospitalier Universitaire de Lille, France (H.H., F.C.).; Department of Neurology, Hospital Vall d’Hebron, Barcelona, Spain (M.O.-G., M.R., J.M.).; Department of Neurology (J.P.M., R.V.), Hospital de Egas Moniz, Centro Hospitalar Lisboa Ocidental, Portugal.; Department of Neuroradiology (J.N.R.), Hospital de Egas Moniz, Centro Hospitalar Lisboa Ocidental, Portugal.; Department of Neurology, Hôpital Civil Marie Curie, Charleroi, Belgium (A.D., F.B.).; Department of Neuroscience and Stroke Program, Bon Secours Mercy Health St. Vincent Hospital, Toledo, OH (O.O.Z., E.L.).; Department of Neurology, Grady Memorial Hospital, Atlanta, GA (D.C.H., M.H.M.).; Department of Radiology, Boston Medical Center, Boston University Chobanian and Avedisian School of Medicine, MA (M.A., M.C.).; Department of Interventional Neuroradiology, Centre Hospitalier de l’Universite de Montreal, Canada (J.R., D.R.).; Department of Neurology, University of Iowa, Iowa City (S.O.-G., J.C.).; Department of Neurology, UTHealth McGovern Medical School, Neurology, Houston, TX (S.A.S., S.S.-M.).; Division of Stroke Prevention and Treatment, Institute of Medicine, University of Tsukuba, Japan (H.Y.).; Institute of Neuroradiology, Faculty of Medicine and University Hospital Carl Gustav Carus, Technische Universität Dresden, Germany (D.P.O.K.).; Division of Stroke Care Unit, National Cerebral and Cardiovascular Center, Suita, Japan (K.T.).; Stroke Center, Kindai University Hospital, Osakasayama, Japan (K.T.).; Division of Interventional Neuroradiology, University of Massachusetts Memorial Medical Center, Worcester (A.S.P.).; Department of Neurology, University of Chicago, IL (J.E.S.).; Department of Neurology, University of Toledo, OH (S.F.Z., M.J., A.C.C.).; Department of Neurology, State University of New York, Upstate Medical University, Syracuse (H.E.M.).; Department of Neurology, Rhode Island Hospital, Providence (L.S., S.Y.).; Department of Neurology, The First Affiliated Hospital of USTC, China (W.H.).; University of Lille, Inserm, CHU Lille, U1172—LilNCog—Lille Neuroscience & Cognition, France (C.C.).

**Keywords:** intracranial hemorrhage, ischemic stroke, middle cerebral artery, thrombectomy, tomography

## Abstract

**BACKGROUND:**

It remains unclear whether outcomes of patients treated with endovascular thrombectomy with large-vessel occlusion and unwitnessed onset of stroke differ from those with witnessed onset in the extended time window.

**METHODS:**

We enrolled patients with anterior circulation large-vessel occlusion (internal carotid artery, M1, or M2 segment of the middle cerebral artery) undergoing endovascular thrombectomy within 6 to 24 hours from the time last seen well, from 2014 to 2022, at 66 sites in Europe, North America, and Asia. Patients with a prestroke modified Rankin Scale score of >3 or age <18 were excluded. We categorized patients by onset mode as witnessed or unwitnessed. The primary outcome was the modified Rankin Scale shift at 90 days. Secondary outcomes were functional independence, a composite of functional independence or return of Rankin to prestroke level, symptomatic intracranial hemorrhage, mortality, and a composite of severe disability or mortality at 90 days. We applied inverse probability of treatment weighting to compare outcomes between the groups.

**RESULTS:**

Of 5098 patients assessed for eligibility, we included 2073, of whom 1760 (84.9%) had unwitnessed onset, and 313 (15.1%) were witnessed. In the univariate comparison (before inverse probability of treatment weighting), 38.8% of the unwitnessed and 45.7% of the witnessed patients achieved functional independence (*P*=0.022). Mortality was 21.6% among unwitnessed and 22.0% among witnessed (*P*=0.847), and symptomatic intracranial hemorrhage rates were 6.6% and 5.8%, respectively (*P*=0.623). The primary outcome (modified Rankin Scale shift) showed no difference comparing unwitnessed to witnessed patients (odds ratio, 1.35 [95% CI, 0.82–2.20]; *P*=0.235) in the inverse probability of treatment weighting. Unwitnessed patients were more likely to achieve functional independence or return of Rankin (1.53 [1.01–2.33]; *P*=0.045). Other secondary outcomes did not differ between the witnessed and unwitnessed patients.

**CONCLUSIONS:**

In the extended time window, unwitnessed patients with large-vessel occlusion undergoing endovascular thrombectomy have at least the same likelihood of favorable outcomes as witnessed patients.

**REGISTRATION:**

URL: https://www.clinicaltrials.gov; Unique identifier: NCT04096248.

The benefit of endovascular thrombectomy (EVT) is well established in the treatment of acute ischemic stroke caused by large vessel occlusion (LVO), also in the extended time window from 6 to 24 hours.^1–8^ Factors that are associated with a favorable outcome include successful recanalization, younger age, initial stroke severity, time to treatment, and the extent of ischemia on baseline imaging.^9–13^

Previous studies have reported variable proportions of patients with witnessed versus unwitnessed stroke onset.^14–17^ Although, in general, up to one-third of patients with stroke have unwitnessed onset,^17^ the percentage is likely higher among patients undergoing EVT in the late window. In the DAWN trial (Clinical Mismatch in the Triage of Wake Up and Late Presenting Strokes Undergoing Neurointervention With Trevo), 88% of patients had an unwitnessed onset of stroke.^18^ Studies addressing the outcomes of patients undergoing EVT with witnessed versus unwitnessed onset are scarce. Based on secondary analyses of the DAWN trial, patients with acute ischemic stroke benefit from EVT regardless of the wake-up, unwitnessed, or witnessed mode of onset.^18^ However, it remains unclear how witnessed versus unwitnessed stroke onset modifies the outcomes in unselected patient cohorts.

The aim of the present secondary analysis of the CLEAR study (CT for Late Endovascular Reperfusion) was to compare outcomes of patients with unwitnessed versus witnessed onset with anterior circulation LVO undergoing EVT between 6 and 24 hours from the time last seen well (TLSW).^19^

## Methods

### Data Availability

Anonymized data are available, based on the local legislation, on reasonable request to the corresponding author.

### Ethics Statement

We used data from the CLEAR study, an investigator-initiated multicenter cohort study following the Strengthening the Reporting of Observational Studies in Epidemiology guidelines.^20^ Each participating site obtained approval for the study from a local institutional review board or ethics committee, following local guidelines and legislation. Written informed consent was waived due to the retrospective design of the study. The anonymized data were centrally collected at Boston Medical Center, Boston. Several authors (T.N.N, L.T., D.S.) and the lead statistician (M.M.Q) had full access to the data.

### Study Population

The study recruited consecutive patients undergoing EVT due to anterior LVO, defined as occlusion in the internal carotid artery or M1 or M2 segment of the middle cerebral artery, 6 to 24 hours from TLSW to groin puncture. The recruitment took place between January 2014 and May 2022 at 66 sites across Europe, North America, and Asia. The study cohort has been described in detail elsewhere.^19^

We excluded patients with age <18 years, missing baseline National Institutes of Health Stroke Scale (NIHSS) score, prestroke modified Rankin Scale (mRS) or Alberta Stroke Program Early Computed Tomography Score (ASPECTS), missing 90-day mRS, missing TLSW or TLSW <6 hours or >24 hours, as well as patients who received only medical management.

We categorized patients based on the mode of onset as witnessed (onset reported to be seen by an observer, such as a family member, or reported by the patient) and unwitnessed (the event was not witnessed, and hence the patient’s TLSW was used as the onset time).

### Outcome Variables

The primary outcome was the 90-day mRS ordinal shift distribution. Secondary outcomes were functional independence (FI), defined as an mRS score of 0 to 2, and FI or return of the mRS to prestroke level (return of Rankin [RoR]) at 90 days. Safety outcomes were symptomatic intracranial hemorrhage (defined by the European Cooperative Acute Stroke Study III: intracranial hemorrhage associated with deterioration in NIHSS score of ≥4 points and the main reason for neurological deterioration),^21^ mortality, and a composite of severe disability or mortality (mRS score of 5–6) at 90 days. Outcomes were prospectively collected as part of the clinical routine by each center.

### Statistical Analysis

We used the Shapiro-Wilk test to evaluate data normality. Data were deemed non-normal and nonparametric. The Mann-Whitney *U* test was used to compare continuous and ordinal variables between groups. Continuous data are reported as median with interquartile range. The Pearson χ^2^ test or Fisher exact test was used to compare categorical variables between the groups.

We performed univariable, multivariable, and inverse probability of treatment weighting (IPTW) logistic regression analyses to evaluate outcomes by type of stroke onset. For the distribution of 90-day mRS (ordinal mRS shift), an ordinal logistic regression model was applied to estimate a 1-point shift toward the lower ordered value, indicating a better outcome. The model was fitted using a cumulative logit link function and ordinal distribution. A logistic regression model was utilized for binary outcomes, employing a logit link function and specifying a binomial distribution. In all analyses, we accounted for clustering by sites using a generalized estimating equation approach. An independence correlation structure was assumed to address the within-site clustering of patients. This independence correlation structure yielded the smallest quasi-likelihood independence criterion value. We computed odds ratios (OR) along with 95% CIs.

For the multivariable and IPTW analyses, the following covariates were selected a priori based on literature as known prognostic factors: age, sex, baseline NIHSS, prestroke mRS, hypertension, diabetes, atrial fibrillation, intravenous thrombolysis, ASPECTS, occlusion site, and TLSW before groin puncture. If TLSW to groin puncture was missing, TLSW to imaging and TLSW to door were used.

We present results from the IPTW logistic regression model as the primary analysis to account for confounding effects. Using a multivariable logistic regression model, we first estimated the probability of unwitnessed onset (PS) conditional on the above covariates. For IPTW, the unwitnessed group received weights of [1/PS], and the witnessed group received weights of (1/[1−PS]). The weights for unwitnessed and witnessed groups were stabilized by replacing the numerator 1 with the proportion of patients with unwitnessed and witnessed onset, respectively. In IPTW, we estimate the average treatment effect (the impact of unwitnessed onset if the entire population had unwitnessed stroke versus if the entire population had witnessed stroke). The balance of covariates after weighting was evaluated using standardized mean differences. A standardized mean difference of <0.15 was considered a good balance.^22^ Crude ORs were calculated using univariable analysis of the weighted population.

#### Interaction Analysis

To determine if baseline characteristics modify the association between witness status and outcomes of ordinal mRS shift and FI or RoR at 90 days, we performed interaction analysis using a multiplicative interaction term in the IPTW model for prespecified baseline characteristics: age (<80 versus ≥80 years), sex, baseline NIHSS (<15 versus ≥15), prestroke mRS (<2 versus ≥2), transfer status, ASPECTS (<7 versus ≥7), intravenous thrombolysis, site of arterial occlusion (internal carotid artery, M1, M2), imaging modality (unenhanced CT, CT perfusion, magnetic resonance imaging), and TLSW to groin puncture (<12 versus ≥12 hours).

#### Additional Analyses

To address potential residual confounding, we conducted a sensitivity analysis to account for imbalances in covariates after weighting by including variables that showed imbalance in the IPTW regression model. Statistical analyses were performed using SAS version 9.4 (SAS Institute, Cary, NC). All tests were 2-sided, and a *P* value of <0.05 was considered statistically significant.

## Results

### Baseline Characteristics

Of 5098 patients in the CLEAR study, 2087 (40.9%) were eligible for the present analysis. After excluding 14 patients due to missing covariate data, the final cohort included 2073 patients, of whom 1760 (84.9%) had unwitnessed, and 313 (15.1%) witnessed onset of symptoms (Figure).

**Figure.**
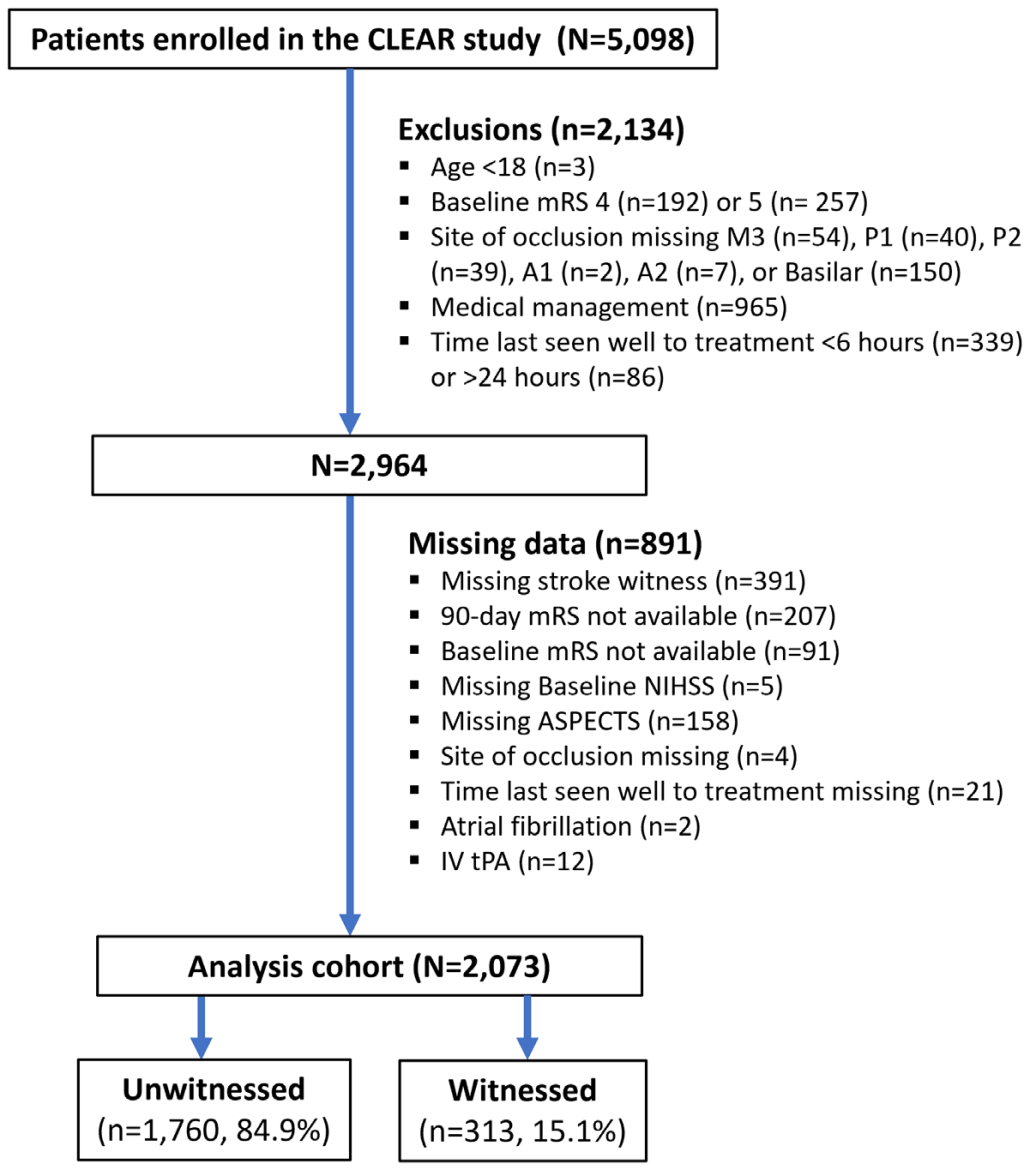
**Patient flowchart.** ASPECTS indicates Alberta Stroke Program Early Computed Tomography Score; CLEAR, CT for Late Endovascular Reperfusion; IV tPA, intravenous tissue-type plasminogen activator; mRS, modified Rankin Scale; and NIHSS, National Institutes of Health Stroke Scale.

Table 1 presents the univariate comparison of baseline characteristics of the study cohort. Unwitnessed patients were older (72 versus 71 years; *P*=0.017), had higher baseline NIHSS (16 versus 13; *P*<0.0001), had fewer transfers (56.1% versus 73.1%; *P*<0.0001), and received intravenous thrombolysis less frequently (17.8% versus 34.5%; *P*<0.0001) than witnessed patients. Unwitnessed patients had lower baseline ASPECTS (8 versus 9; *P*=0.002). The TLSW to imaging and treatment was longer for unwitnessed than witnessed patients (12.2 hours versus 8.3 hours; *P*<0.0001, and 11.0 hours versus 7.0 hours, *P*<0.0001, respectively). Unwitnessed patients compared with witnessed patients more often achieved successful reperfusion, defined as modified Treatment in Cerebral Infarction score ≥2b (85.8% versus 79.1%; *P*=0.003). However, when considering near complete or complete reperfusion (modified Treatment in Cerebral Infarction score ≥2c), there was no difference between the unwitnessed and witnessed patients (54.9% versus 49.8%; *P*=0.098).

**Table 1.**
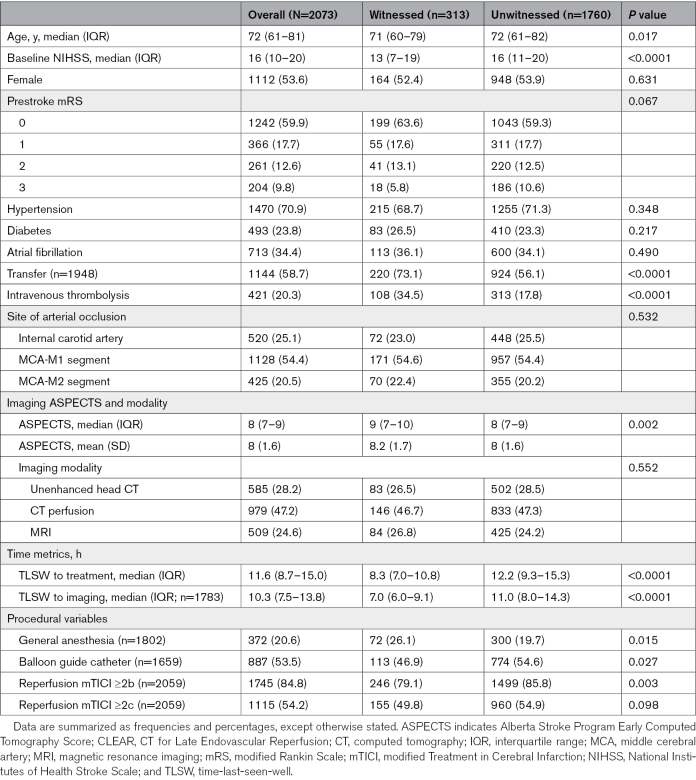
Baseline Characteristics and Metrics of CLEAR Patients in the 6-to-24-Hour Window Stratified by Stroke Witness

### Outcome Analysis

The median mRS at 90 days was 3 (interquartile range, 2–5) among unwitnessed and 3 (interquartile range, 1–5) among witnessed patients (*P*=0.057). Of the unwitnessed and witnessed patients, 683 (38.8%) and 143 (45.7%) achieved FI (*P*=0.022), respectively. The mortality rate was 21.6% among unwitnessed and 22.0% among witnessed (*P*=0.847), and the symptomatic intracranial hemorrhage rate was 6.6% and 5.8%, respectively (*P*=0.623; Table 2).

**Table 2.**
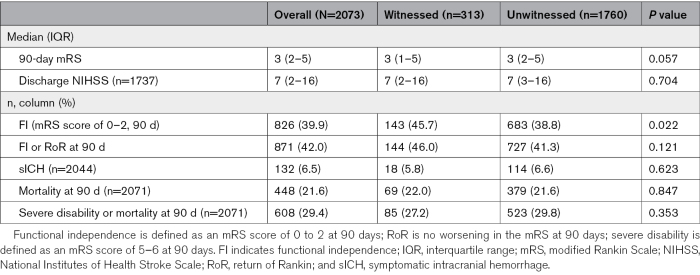
Clinical Outcomes by Stroke Witness

In the IPTW regression, the primary outcome of ordinal shift on the mRS scale at 90 days did not show a statistically significant difference between unwitnessed and witnessed patients (OR, 1.35 [95% CI, 0.82–2.20]; *P*=0.235). Unwitnessed patients were more likely to achieve FI or RoR at 90 days than witnessed (OR, 1.53 [95% CI, 1.01–2.33]; *P*=0.045). Other outcome measures (FI at 90 days, symptomatic intracranial hemorrhage, mortality at 90 days, or composite of severe disability or mortality at 90 days) did not show a significant difference between groups. The complete results from the univariable, multivariable, and IPTW models are displayed in Table 3.

**Table 3.**
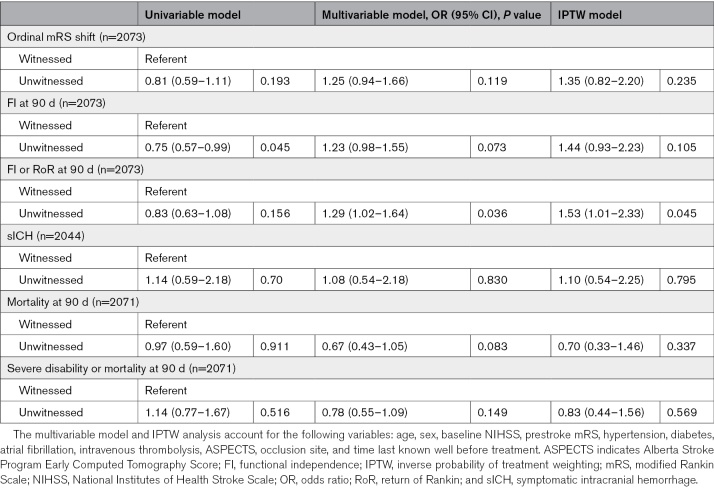
Univariable, Multivariable, and IPTW Logistic Regression Analyses of Outcomes by Stroke Witness

Sex, occlusion site, and TLSW before treatment showed potential imbalance between the groups after the IPTW, defined as a standardized mean difference >0.15 (Table S1). Hence, we performed a sensitivity analysis by including these variables in the IPTW regression model. The result remained unchanged, that is, unwitnessed patients were more likely to achieve FI or RoR at 90 days compared with witnessed patients (OR, 1.51 [95% CI, 1.02–2.22]; *P*=0.038), while the primary outcome of ordinal mRS shift did not show a difference between the groups (Table S2).

### Interaction Analysis

The interaction analysis suggested that males and transferred patients had a higher likelihood of favorable outcomes if their onset of symptoms was unwitnessed. However, no interaction was observed for FI or RoR (Table 4). There was no interaction in ordinal mRS shift or FI or RoR outcomes between witnessed and unwitnessed patients according to age, prestroke mRS, baseline ASPECTS, use of intravenous thrombolysis, site of arterial occlusion, imaging modality, or TLSW.

**Table 4.**
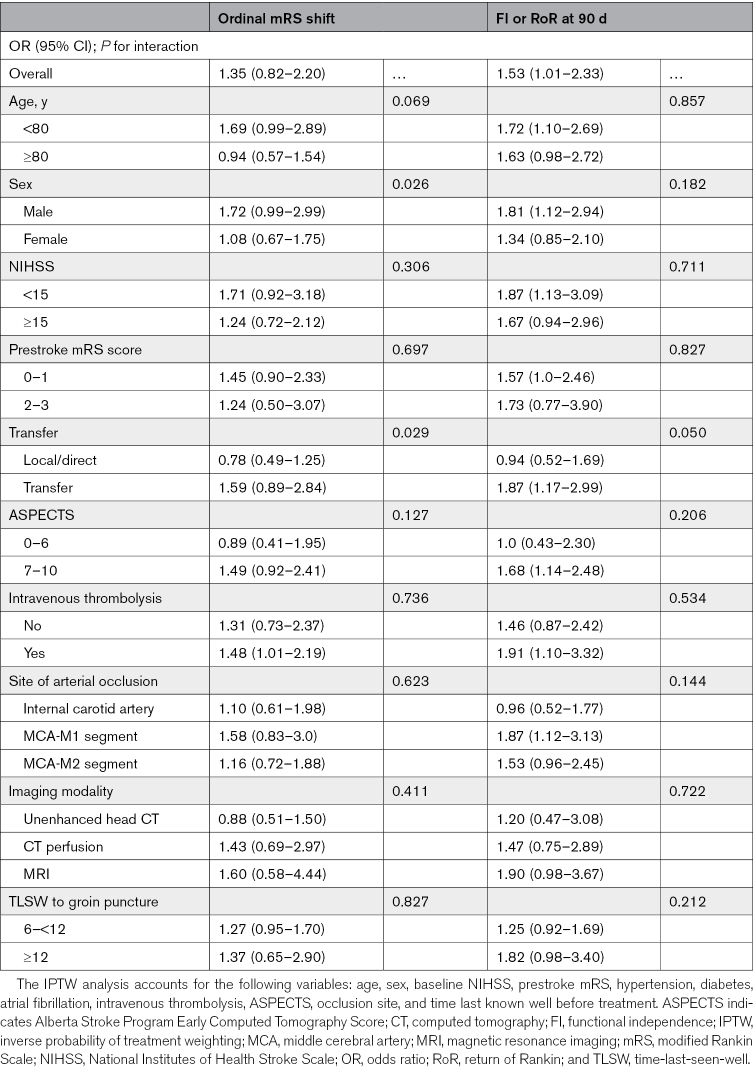
IPTW Logistic Regression of Ordinal mRS Shift and FI or RoR at 90 Days: Interaction of Stroke Witness With Baseline Characteristics on Outcomes With Witnessed as the Reference Category

## Discussion

We studied how unwitnessed versus witnessed onset of stroke modifies functional and safety outcomes in our large, multicenter study cohort of patients with stroke with LVO undergoing EVT in the extended time window from 6 to 24 hours from TLSW. The vast majority of patients had an unwitnessed stroke onset (85%). The primary outcome of the ordinal mRS shift at 90 days did not show a difference between unwitnessed and witnessed patients, whereas unwitnessed patients were more likely to achieve the secondary outcome of FI or RoR at 90 days compared with witnessed patients. Other secondary outcomes, including FI and safety outcomes, including symptomatic intracranial hemorrhage and mortality, did not differ between unwitnessed and witnessed patients.

Although only a small proportion of patients had a witnessed onset, the distribution of stroke onset types in our cohort is comparable to other late window EVT trials, including the DAWN and MR CLEAN-LATE (Multicenter Randomized Clinical Trial of Endovascular Treatment of Acute Ischemic Stroke in the Netherlands for Late Arrivals) trials, which included anterior LVO patients with a similar time window, that is, 6 to 24 hours from TLSW. In our study, 15% of patients had witnessed stroke onset compared with 12% in DAWN and 11% in MR CLEAN-LATE.^18,23^ In the DEFUSE 3 trial (Endovascular Therapy Following Imaging Evaluation for Ischemic Stroke 3), which randomized patients into EVT or standard medical care, 36% of patients had a witnessed onset within 6 to 16 hours from TLSW. In the Chinese TRACE-III trial (Tenecteplase Reperfusion Therapy in Acute Ischemic Cerebrovascular Events-III), which randomized patients with anterior LVO into intravenous thrombolysis or standard medical care when access to EVT was not available, 57% of participants had a witnessed onset (within the 4.5–24-hour time window).^2,24^ Patients with more severe stroke symptoms tend to seek help faster than patients with minor symptoms.^25^ Whether or not a patient is categorized as witnessed or unwitnessed can also vary based on the depth to which a history is obtained when looking for potential witnesses. Additionally, cultural or access differences may lead to a longer time to treatment in general, which could increase the proportion of witnessed patients within the extended time window.^26^

The witnessed patients can be categorized as being in the extended time window with high certainty, as the precise onset time of symptoms is known. In contrast, the time window for unwitnessed patients is uncertain; symptoms may have begun at any point between TLSW and symptom recognition, meaning that some may have actually fallen within the early window (<6 hours), and, in general, time windows might be shorter in unwitnessed than witnessed patients. Likely, those who are most likely to benefit from EVT in the extended time window are either slow progressors or, in the case of an unwitnessed stroke, those in whom the actual time window might be shorter.^27,28^ While most of our patients underwent some form of advanced imaging—47.2% with CT perfusion and 24.6% with magnetic resonance imaging—we did not have specific inclusion criteria regarding salvageable brain tissue or the extent of the ischemic changes. Further, without more detailed imaging parameters, we were unable to conduct further analyses of the imaging profiles that could have provided clues on the time metrics of the stroke onset.

In our study, we did not have information on the wake-up stroke (WUS) status available. However, the unwitnessed group also included the patients with WUS. Previous studies suggest that in patients with WUS, symptom onset often occurs closer to the time of awakening rather than TLSW, as inferred from imaging profiles.^29–32^ Analyses from the DAWN trial demonstrated that patients benefited from EVT in the extended time window regardless of onset type—witnessed, WUS, or unwitnessed non-WUS.^18^ A substudy of the DAWN trial revealed that patients with WUS treated successfully with EVT showed a significant reduction in the likelihood of FI over time (from TLSW to treatment or reperfusion). In contrast, this time interval did not significantly impact outcomes in patients with witnessed or non-witnessed strokes.^32^ Previous studies have suggested that patients with WUS and unwitnessed non-WUS patients might also have different characteristics due to, for example, cardiovascular circadian variation or clinical presentation; unwitnessed non-WUS patients often have more severe symptoms.^16,33–35^ Thus, slightly better outcomes of unwitnessed patients compared with witnessed strokes in our study could at least, in part, be due to special characteristics of the patients with WUS.

In our cohort, both sex and transfer status showed interaction with stroke witness status regarding the primary outcome of mRS shift. Although both males and females are reported to benefit equally from EVT, differences exist in baseline characteristics and stroke subtypes.^9,36,37^ Female patients more often live alone, which can cause delays in seeking treatment.^38^ Although neither sex showed a significant difference between unwitnessed and witnessed onset on the primary outcome, males were slightly more likely to benefit from unwitnessed status. One possible explanation for the sex difference is that females are more often unwitnessed because they live alone and are unable to seek treatment themselves. Previous studies have shown that transferred patients experience longer treatment delays but a similar likelihood of good functional outcomes.^39,40^ In our cohort, patients who were transferred were more likely to benefit from being unwitnessed. Further studies should investigate how transfer status affects outcomes in various patient subgroups.

Strengths of our study include the large, real-world patient cohort, which provides a broad geographic representation of individuals from Europe, North America, and Asia. However, we also acknowledge several limitations. The retrospective design of the study introduces the potential for selection bias, which could limit the generalizability of our findings. Additionally, the availability of variables was restricted by the nature of the retrospective data collection. For instance, the determination of witness versus unwitnessed status was left to the treating physician’s judgment. Moreover, due to the retrospective design, we did not have a time point for symptom recognition available. Thus, we were unable to analyze the intervals between TLSW and symptom onset. Another limitation is the missing data: 30% of patients otherwise eligible had incomplete information regarding either exposure, outcome data, or covariate data and were consequently excluded from the analysis (Figure). Our cohort did not have WUS status available. Further studies are of interest to evaluate how the WUS versus non-WUS status modifies the outcomes among patients with unwitnessed stroke undergoing EVT in the extended time window.

In conclusion, the results from our large patient cohort show that patients who experience unwitnessed strokes have at least the same likelihood of favorable outcomes following EVT as witnessed patients. The safety outcomes were comparable.

## ARTICLE INFORMATION

### Sources of Funding

The study was funded by Medtronic Inc.

### Disclosures

Dr Nguyen reports compensation from Genentech for other services and compensation from Kaneka for other services. Dr Nogueira reports stock options in Cerebrotech; Reist/Q?Apel Medical, RapidPulse, Brain4Care, Viseon Inc, Truvic, Quantanosis AI, and Piraeus Medical; compensation for consultant services from Cerebrotech, Astrocyte, Boehringe, Hybernia, Philips, and Shanghai Wallaby; and Synchron for data and safety monitoring services. Dr Nagel reports stock options from Brainomix, and compensation from Böhringer Ingelheim, Bristol-Myers Squibb, and CSL Behring for consulation services. Dr Henon reports compensation from Novartis Pharma for other services and grants from Sanofi-Aventis U.S. LLC. Dr Olive-Gadea reports other intellectual property. Dr Haussen reports compensation from Medtronic for consultant services and stock options in Motif. Dr Ortega-Gutierrez reports grants from Stryker; employment by Carver College of Medicine—University of Iowa; grants from Siemens; stock options in Active Motif; grants from National Institutes of Health; compensation from Stryker and Medtronic for consultant services; grants from PCORI, methinks, and National Institutes of Health; stock holdings in Eureka Therapeutics; and stock options in BrainFlow. Dr Sheth reports compensation from University of Iowa Hospitals & Clinics for other services; compensation from Route 92 Medical Inc for consultant services; compensation from Bayer HealthCare Pharmaceuticals Inc for other services; compensation from phenox Inc. for data and safety monitoring services; compensation from Medtronic for consultant services; compensation from Perfuze for other services; Inc for consultant services. Dr Ribo reports compensation from Sensome for data and safety monitoring services; compensation from Vesalio for consultant services; compensation from Rapid Pulse for consultant services. Dr Puri reports compensation from Johnson and Johnson, Medtronic, Route 92 Medical Inc, and MicroVention Inc for consultant services. Dr Siegler reports compensation from Novartis for consultant services. Dr Lin reports compensation from Imperative Care Inc for consultant services. Dr Wouters reports compensation from Bayer Healthcare for consultant services. Dr Herweh reports consultancy to Brainomix Ltd (Oxford, UK). Dr Möhlenbruch reports compensation from Johnson and Johnson and Siemens for consultant services. Dr Strbian reports employment by Helsingin ja Uudenmaan Sairaanhoitopiiri; compensation from AstraZeneca AB for consultant services and grants from Boehringer Ingelheim.

The other authors report no conflicts.

### Supplemental Material

Tables S1–S2

STROBE Statement

## Supplementary Material


