# Supplementary material for: Outcomes of Witnessed Versus Unwitnessed Patients With Stroke After Endovascular Therapy in the Extended Time Window
Source: Stroke. 2025 Nov 12;57(2):362–70. doi: 10.1161/STROKEAHA.125.052355 (PMC12829500; doi:10.1161/STROKEAHA.125.052355)
Supplement: Supplementary file 1 [file str-57-362-s001.pdf]

**Supplemental material to: Outcomes of witnessed versus unwitnessed stroke patients after endovascular therapy in the extended time window**

**Table S1. Baseline characteristics before and after the inverse probability of treatment weighting by stroke witness.**

| | Unweighted | | | IPTW-adjusted | | |
|---|---|---|---|---|---|---|
| | Witnessed (N=313) | Unwitnessed (N=1,760) | SMD | Witnessed | Unwitnessed | SMD |
| Age (years), median (IQR) | 71 (60-79) | 72 (61-82) | -0.15 | 71 (59-79) | 72 (61-81) | -0.12 |
| Baseline NIHSS, median (IQR) | 13 (7-19) | 16 (11-20) | -0.35 | 14 (9-19) | 16 (10-20) | -0.11 |
| Female | 52.4 | 53.9 | 0.03 | 44.3 | 53.3 | 0.18 |
| Prestroke mRS | | | 0.18 | | | 0.10 |
| 0 | 63.6 | 59.3 | | 62.3 | 59.9 | |
| 1 | 17.6 | 17.7 | | 18.6 | 17.8 | |
| 2 | 13.1 | 12.5 | | 11.9 | 12.5 | |
| 3 | 5.8 | 10.6 | | 7.3 | 9.9 | |
| Hypertension | 68.7 | 71.3 | -0.06 | 68.8 | 70.9 | -0.04 |
| Diabetes | 26.5 | 23.3 | 0.07 | 23.0 | 23.9 | -0.02 |
| Atrial fibrillation | 36.1 | 34.1 | 0.04 | 32.5 | 34.5 | -0.04 |
| Intravenous thrombolysis | 34.5 | 17.8 | 0.39 | 19.3 | 20.4 | -0.03 |
| Site of arterial occlusion | | | 0.07 | | | 0.17 |
| Internal carotid artery | 23.0 | 25.5 | | 18.1 | 24.9 | |
| MCA-M1 segment | 54.6 | 54.4 | | 59.0 | 54.4 | |
| MCA-M2 segment | 22.4 | 20.2 | | 22.9 | 20.7 | |
| **Imaging ASPECTS and modality** | | | | | | |
| ASPECTS, median (IQR) | 9 (7-10) | 8 (7-9) | 0.16 | 8 (7-9) | 8 (7-9) | -0.03 |
| **Time Metrics (hours)** | | | | | | |
| TLSW to treatment, median (IQR) | 8.3 (7.0-10.8) | 12.2 (9.3-15.3) | -0.71 | 14.0 (8.5-19.4) | 11.8 (8.9-14.9) | 0.35 |

Abbreviations: IPTW= inverse probability of treatment weighting; SMD= standardized mean difference; IQR= interquartile range; mRS=modified Rankin Scale; ASPECTS=Alberta Stroke Program Early CT Score; MCA=middle cerebral artery; TLSW= time-last-seen-well.

Note: The IPTW analysis accounts for the following variables: age, sex, baseline NIHSS, prestroke mRS, hypertension, diabetes, atrial fibrillation, intravenous thrombolysis, ASPECTS, occlusion site, and time last known well prior to treatment.

**Table S2. IPTW logistic regression of ordinal mRS shift and FI or RoR at 90 days by stroke witness: controlling for residual confounding (factors with SMD >0.15 post-weighing).**

| | Ordinal mRS shift | | FI or RoR at 90 days | |
|---|---|---|---|---|
| | OR (95% CI), *P* | | | |
| | *Core model* | | | |
| Witnessed | referent | | | |
| Unwitnessed | 1.35 (0.82-2.20) | 0.235 | 1.53 (1.01-2.33) | 0.045 |
| | *Adding sex, occlusion site, and time last known well prior to treatment.* | | | |
| Witnessed | referent | | | |
| Unwitnessed | 1.32 (0.84-2.07) | 0.223 | 1.51 (1.02-2.22) | 0.038 |

Abbreviations: IPTW, inverse probability of treatment weighting; mRS=modified Rankin Scale; FI= functional independence; RoR= Return of Rankin; OR, odds ratio; CI, confidence interval; N=total number of patients.

Note: The core model accounts for the following variables: age, sex, baseline NIHSS, prestroke mRS, hypertension, diabetes, atrial fibrillation, intravenous thrombolysis, ASPECTS, occlusion site, and time last known well prior to treatment.
